# Impact of the double deletion ΔG242-T243 in KPC-2 in the effectiveness of ceftazidime-avibactam and imipenem-relebactam

**DOI:** 10.1128/aac.01915-24

**Published:** 2025-05-05

**Authors:** Florencia Brunetti, Gabriel Gutkind, Lin Gao, Shozeb Haider, Robert A. Bonomo, Pablo Power

**Affiliations:** 1 Universidad de Buenos Aires, Instituto de Investigaciones en Bacteriología y Virología Molecular (IBaViM), Facultad de Farmacia y Bioquímica, Buenos Aires, Argentina; 2 Consejo Nacional de Investigaciones Científicas y Técnicas (CONICET) 62873, Buenos Aires, Argentina; 3 UCL School of Pharmacy 371646 , London, United Kingdom; 4 Prince Fahad Bin Sultan Chair for Biomedical Research (PFSCBR), University of Tabuk 125900, Tabuk, Saudi Arabia; 5 UCL Centre for Advanced Research Computing 392547 , London, United Kingdom; 6 Research Service, Louis Stokes Cleveland Department of Veterans Affairs Medical Center 465630, Cleveland, Ohio, USA; 7 Department of Medicine, Case Western Reserve University School of Medicine 12304, Cleveland, Ohio, USA; 8 Clinician Scientist Investigator, Louis Stokes Cleveland Department of Veterans Affairs Medical Center 20083, Cleveland, Ohio, USA; 9 Department of Molecular Biology and Microbiology, Case Western Reserve University School of Medicine 12304, Cleveland, Ohio, USA; 10 Department of Pharmacology, Case Western Reserve University School of Medicine, Cleveland, Ohio, USA; 11 Department of Biochemistry, Case Western Reserve University School of Medicine, Cleveland, Ohio, USA; 12 Department of Proteomics and Bioinformatics, Case Western Reserve University School of Medicine, Cleveland, Ohio, USA; 13 CWRU-Cleveland VAMC Center for Antimicrobial Resistance and Epidemiology (Case VA CARES), Cleveland, Ohio, USA; University of Fribourg, Fribourg, Switzerland

**Keywords:** KPC-14, CZA, DBO, *Klebsiella pneumoniae*, avibactam, relebactam

## Abstract

Combinations of β-lactam-diazabicyclooctane inhibitors (DBOs) like ceftazidime-avibactam (CZA) and imipenem-relebactam (IMR) have shown efficacy in treating KPC-2-producing *Klebsiella pneumoniae*. However, CZA-resistant *K. pneumoniae* strains have been identified, often linked to substitutions and/or insertions/deletions in three different loops of KPC: (i) the Ω-loop region (amino acids 164–179), (ii) the 237–243 loop; and (iii) the 266–275 loop. This study investigates the impact of the double deletion ΔG242-T243 present in KPC-14. Our results demonstrate that the lower effectiveness of CZA against KPC-14 can be explained by both increased hydrolysis of ceftazidime and a lower affinity and acylation rate by avibactam. In contrast, the IMR combination was efficient in restoring susceptibility to the KPC-14 producing-clone. Although we also observed a lower affinity and acylation rate for relebactam in KPC-14, this reduction in affinity was accompanied by a loss in the carbapenemase activity, finally resulting in an IMR susceptibility phenotype for KPC-14. Expansion of the substrate profile of KPC-14 toward ceftazidime is associated with a trade-off for carbapenems, other penicillins, and cephalosporins, as well as a higher inhibition by clavulanic acid compared to KPC-2. This study provides a better understanding of how deletions in the 237–243 loop affect the effectiveness of novel DBO-combinations and supports the hypothesis that these mutations result in CZA resistance by other different biochemical mechanisms than mutations in the Ω-loop.

## INTRODUCTION

**Fig 1:**
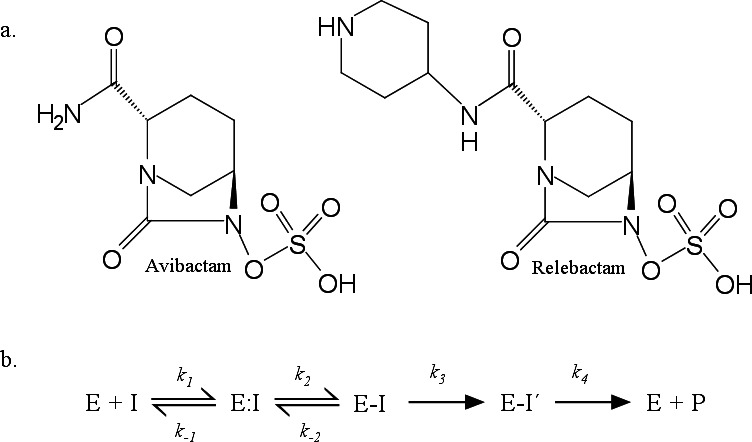
(**a**) Chemical structure of avibactam (AVI) and relebactam (REL). (**b**) Kinetic model proposed for the interaction of KPC-2 with AVI and REL (3). In this model, E and I represent the enzyme and the DBO inhibitors, respectively. E:I denotes the noncovalent complex, while E-I represents the enzyme acylated with AVI or REL. Initially, the enzyme is reversibly acylated by the DBO inhibitor. However, for KPC-2, a slow hydrolytic pathway involving the loss of the sulfate and an imine hydrolysis (E-I’) was proposed for both AVI and REL. The final step results in the deacylated enzyme and an oxopiperidine product (P).

*Klebsiella pneumoniae* producing KPC-2, one of the most widespread serine carbapenemases, is known as one of the most relevant clinical threats (1). Combinations of β-lactam-diazabicyclooctane inhibitors (DBOs) such as ceftazidime-avibactam (CZA) and imipenem-relebactam (IMR) have proven to be successful in treating infections by isolates harboring KPC-2 (2–5). The proposed mechanism for the reversible and efficient inhibition of KPC-2 by avibactam (AVI) and relebactam (REL) is presented in Fig. 1. For AVI, a slow two-step hydrolytic mechanism was proposed after the acylation step of KPC-2 (3). Even if KPC-2 was not initially reported that could hydrolyze REL (4), subsequent crystallographic studies revealed that REL is also desulfated by KPC-2 and the KPC D179N variant, but at a slower rate compared to avibactam (6, 7). It was, therefore, hypothesized that the larger R1 group in REL would sterically impair a favorable rotation of the piperidine ring for desulfation (6, 7).

Since the FDA’s approval of CZA in 2015, this combination is considered a viable treatment option for carbapenem-resistant *Enterobacterales* (CRE) producing KPC enzymes (5, 8). However, CZA-resistant isolates have rapidly emerged associated with amino acid substitutions and/or insertions/deletions in three “hotspots” in the KPC β-lactamase: (i) the Ω-loop region (amino acids 164–179); (ii) the 237–243 loop; and (iii) the 266–275 loop (9). Each loop seems to tolerate different types of amino acid changes. To summarize, in the Ω loop substitutions, deletions, and insertions are described. In contrast, only substitutions and deletions at the 237–243 loop are reported, and only substitutions and insertions at the 266–275 loop have been recovered so far in clinical isolates (9).

Of all KPC β-lactamases, the variants with substitutions in the Ω-loop that confer resistance to CZA have been extensively studied biochemically (10–15). Among the substitutions most frequently found, those in the D179 residue are the most widespread (16, 17). Generally, substitutions in this “hotspot” result in a lower *K*_M_ and lower turnover rate for ceftazidime (CAZ), accompanied by a variable impact on the interaction with avibactam (10–12). Particularly for the D179Y variant, pre-steady-state kinetics showed that the decrease in *K_M_* values is associated with a shift in the rate-limiting step of ceftazidime hydrolysis from acylation to deacylation, where the acylated enzyme may act as a β-lactam trap for this cephalosporin (13).

Structural studies of R164 and D179 variants suggest that this kinetic effect might be due to the loss of important noncovalent bonds that stabilize the Ω-loop motif, leading to a more flexible loop that can accommodate the bulky ceftazidime (14, 15). However, a more flexible Ω-loop could compromise the positioning of relevant residues involved in the deacylation step, like E166 and N170, that can result in the mentioned “β-lactam trap” where substrates are not hydrolyzed efficiently (10, 14, 15). The role of N170 has also been highlighted as a result of changes in the hydrogen bond network in the Ω-loop (15). Therefore, the structural changes that produce lower *K_M_* and turnover rate for CAZ could also affect the proper interactions between KPC and AVI (10, 14, 15). This could be correlated with the lower affinity or slower acylation rate for avibactam reported for the D179Y substitution (11). For D179 variants, residual hydrolysis of CAZ was observed, and crystallographic and computational analyses using accelerated rare-event sampling well-tempered meta-dynamics simulations suggested that a substrate-assisted catalysis mechanism could occur (15, 18).

As for the other two hotspots, previous reports described the kinetic behavior of different KPC variants (19–24). However, there is insufficient data regarding the actual impact of these mutations on the interaction with AVI and REL inhibitors. KPC-14 is a variant harboring a double deletion (ΔG242-T243) reported to elevate MIC values for CZA in some clinical isolates (25–27), and interestingly, even in microorganisms isolated before the introduction of CZA usage (28). Since this deletion is located at the 237–243 loop, we hypothesize that the biochemical mechanism conferring the CZA-resistant phenotype differs from the mechanism previously proposed for KPC variants with changes in the Ω-loop.

In this study, we investigate the biochemical impact of the double deletion ΔG242-T243 present in the KPC-14 variant, focusing on its hydrolytic profile towards β-lactams and the efficacy of the last resort DBO combinations CZA and IMR.

## RESULTS AND DISCUSSION

### Evaluation of resistance and kinetic profile conferred by KPC-14

The differential resistance phenotype conferred by KPC-14 as compared to KPC-2 was evaluated by determining the minimum inhibitory concentration (MICs) against β-lactams and combinations with β-lactamase inhibitors (Table 1).

**TABLE 1:** Minimum inhibitory concentrations (μg/ml) of *E. coli* TOP10F´ recombinant clones

Antibiotic	*E. coli* TOP10F´	Coli TOP10F´/pMBLe	pMBLe/KPC-2	pMBLe/KPC-14
Ampicillin	2	2	1,024	64
Ampicillin-Sulbactam	2/1	2/1	64/32	4/2
Cephalothin	8	8	256	64
Ceftriaxone	≤0.25	≤0.25	8	8
Ceftriaxone-Clavulanate	0.06/0.03	0.06/0.03	8/4	2/1
Ceftriaxone-Avibactam	0.03/4	0.03/4	0.06/4	0.25/4
Ceftriaxone-Relebactam	0.03/4	0.03/4	0.12/4	1/4
Ceftazidime	0.12	0.12	2	32
Ceftazidime - Avibactam	0.06/4	0.06/4	0.5/4	8/4
Cefepime	≤0.25	≤0.25	1	1
Aztreonam	≤0.25	≤0.25	16	8
Imipenem	0.12	0.12	2	0.25
Imipenem-Relebactam	0.12/4	0.12/4	0.12/4	0.25/4
Meropenem	≤0.03	≤0.03	1	≤0.03

The KPC-14 producing recombinant clone displayed reduced MICs (two or more dilutions) for ampicillin, ampicillin-sulbactam, cephalothin, and ceftriaxone-clavulanate compared to KPC-2. Differences in MIC values were not observed for ceftriaxone and cefepime. Aztreonam MICs differed only by one dilution. On the other hand, *bla*_KPC-14_ expression led to higher MIC values for ceftazidime and its combination with avibactam (16-fold higher for both). Unlike ceftazidime-avibactam (CZA), imipenem, meropenem, and the imipenem-relebactam (IMR) combination rendered MIC values within the susceptibility range in the recombinant clone harboring KPC-14.

These MIC values suggest that KPC-14 has a modified hydrolytic profile compared to KPC-2. To support this observation, we determined the kinetic parameters of both enzymes to compare the effects of the ΔG242-T243 mutation on the hydrolysis of various β-lactams (Table 2). Deletion present in KPC-14 negatively affects the hydrolytic efficiency (*k*_cat_*/K*_M_) of most of the tested penicillins, cephalosporins, and carbapenems, except for ceftazidime and cefepime. The most dramatic changes in *k*_cat_*/K*_M_ values were observed for ampicillin, imipenem, and meropenem, being 24-, 11-, and 1,000-fold lower, respectively.

**TABLE 2:** Kinetic parameters comparison of β-lactams hydrolysis

	KPC-2			KPC-14		
β-Lactam substrate	*K*_M_(µM)	*k*_cat_ (s^−1^)	*k*_cat_/*K*_M_ (µM^−1^.s^−1^)	*K*_M_ (µM)	*k*_cat_ (s^−1^)	*k*_cat_/*K*_M_ (µM^−1^.s^−1^)
Ampicillin	537 ± 87	115 ± 8	0.22 ± 0.04	95 ± 10	0.83 ± 0.02	0.009 ± 0.001
Piperacillin	97 ± 12	29 ± 1	0.30 ± 0.04	61 ± 9	3.0 ± 0.1	0.046 ± 0.007
Cephalothin	159 ± 15	56 ± 2	0.36 ± 0.04	94 ± 12	16 ± 1	0.17 ± 0.02
Cefuroxime	319 ± 48	85 ± 8	0.27 ± 0.05	217 ± 31	34 ± 3	0.16 ± 0.03
Ceftriaxone	267 ± 29^*a*^	52 ± 7	0.194 ± 0.006	31 ± 5	4 ± 0.3	0.14 ± 0.03
Ceftazidime	1,319 ± 145^a^	0.9 ± 0.1	(8.3 ± 0.4) × 10^−4^	242 ± 30	5.5 ± 0.4	0.022 ± 0.003
Cefepime	59 ± 5	1.31 ± 0.04	0.022 ± 0.002	7 ± 1	0.61 ± 0.03	0.080 ± 0.012
Imipenem	120 ± 9	15.0 ± 0.4	0.12 ± 0.01	0.55 ± 0.02^*a*^	(5.93 ± 0.02) × 10^−3^	0.0109 ± 0.0004
Meropenem	31 ± 2	2.85 ± 0.06	0.091 ± 0.006	10 ± 1^*a*^	(8.8000 ± 0.0001) × 10^−4^	(8.9 ± 0.9) × 10^−5^

^*a*^ Parameters were determined with nitrocefin used as reporter in competitive assays.

For penicillins, a greater decrease in ampicillin hydrolysis was observed compared to piperacillin. Although KPC-14 exhibits higher affinity (*K*_M_) for both substrates compared to KPC-2, the reduction in hydrolytic efficiency for both penicillins can be attributed to lower turnover rates (*k*_cat_).

Regarding cephalosporins, KPC-14 hydrolyzes cephalothin with half the efficiency (due to a lower *k*_cat_) compared to KPC-2, which is similar to the *k*_cat_/*K*_M_ for cefuroxime. For ceftriaxone, although KPC-14 displays higher affinity and lower turnover value (*k*_cat_), the ratio between these parameters yielded a similar hydrolytic efficiency compared to KPC-2. In contrast, the double deletion in KPC-14 leads to a 30-fold increase in the ceftazidime hydrolytic efficiency and 3-fold for cefepime. In the case of ceftazidime, the increase in *k*_cat_*/K*_M_ is attributed to a fivefold increase in substrate affinity (*K*_M_) and a sixfold increase in the *k*_cat_ value. For cefepime, the slightly higher efficiency compared to KPC-2 is primarily due to increased affinity (lower *K_M_*) for this cephalosporin.

The mutation present in KPC-14 negatively impacts carbapenems hydrolysis. Even if KPC-14 displayed lower *K*_M_ values (higher affinity), a more significant decrease in *k*_cat_ values for both imipenem and meropenem is the main driver for the final phenotypic effects. In fact, *k*_cat_ values with imipenem and meropenem are so low that they may indicate an actual loss of carbapenemase activity in the KPC-14 variant.

The results presented so far indicate that the deletion ΔG242-T243 harbored by the KPC-14 variant produces a modified enzymatic profile compared to KPC-2. Considering this kinetic profile alongside the phenotypic data for the KPC-14-producing clone, it is observed that antibiotics with lower hydrolytic efficiency also yielded lower MIC values compared to KPC-2. For ceftriaxone, both enzymes exhibited similar hydrolytic efficiency and MIC values for their respective clones.

Regarding ceftazidime, the higher hydrolysis of this substrate correlated with a greater MIC value in the KPC-14 producing isolate. The increased hydrolytic rate for ceftazidime has also been reported for other KPC variants with mutations in the 237–243 loop (19, 29). Therefore, the effect of amino acid changes in this loop on the hydrolysis of ceftazidime appears to differ from what has been observed for KPC variants with substitutions in the ꭥ-loop, which exhibit lower *K_M_* values but lower turnover rate for ceftazidime (10–15).

The 30-fold increase in hydrolytic activity towards ceftazidime is accompanied by a loss of carbapenemase activity and reduced hydrolysis of other substrates such as penicillins and some cephalosporins. This trade-off, associated with the expansion of the substrate spectrum to ceftazidime, has been reported not only in other KPC variants (15) but also in other class A β-lactamases such as TEM, CTX-M, and PER (30–32).

Finally, the observed changes in the kinetic profile of KPC-14 in this study correlate with the previously reported findings of Oueslati et al. (20) for this variant. They also described a 40-fold increase in hydrolytic efficiency for CAZ (comparable to the magnitude observed in our work), and loss of carbapenemase activity, reflected in a 1,000-fold reduction in imipenem turnover, similar to the 2,500-fold decrease that we detected.

### The double deletion in KPC-14 produces a reduction in the affinity and acylation rate by avibactam and relebactam

To better understand the increased MIC value for ceftazidime-avibactam observed in the KPC-14 recombinant clone without changes in susceptibility towards imipenem-relebactam, we obtained the inhibitory parameters of both DBOs inhibitors for KPC-14 and KPC-2 (Table 3).

**TABLE 3:** Inhibition kinetic parameters comparison for avibactam and relebactam

	Avibactam		Relebactam	
	*K*_i app_ (µM)	*k*_2_/*K* (M^−1^ s^−1^)	*K*_i app_ (µM)	*k*_2_/*K* (M^−1^ s^−1^)
KPC-2	0.060 ± 0.004	25,000 ± 20	0.060 ± 0.0007	9,100 ± 109
KPC-14	7.8 ± 0.5	125 ± 4	22.9 ± 0.9	65 ± 2

The inhibition parameters determined for KPC-14 showed that this variant has a lower affinity for both DBOs compared to KPC-2. The decreased affinity for these inhibitors was more pronounced for REL than for AVI (*K*_i app_ = 380 and 130-fold higher, respectively). Furthermore, the deletion in KPC-14 not only affects the affinity but also has a negative impact on the acylation rates (*k*_2_/*K*) of both inhibitors. The *k_2_/K* values indicate that the acylation of KPC-14 by REL and AVI occurs at a considerably slower rate compared to KPC-2, with a reduction of 140-fold and 200-fold for REL and AVI, respectively.

These biochemical characteristics provide insights into the observed phenotypic resistance. The MIC values demonstrated that CZA is not efficient in inhibiting the *in vitro* growth of the KPC-14 producing clone, which correlates with the decrease in affinity and acylation rates for this inhibitor.

Notably, while the affinity and acylation rate of relebactam are also impaired in KPC-14, the deletion in this variant simultaneously leads to a significant reduction in the hydrolysis of imipenem. The sum of both biochemical features may result in a phenotype of susceptibility to carbapenems and IMR, in which the decreased relebactam inhibition is offset by increased imipenem efficacy *in vivo*.

Moreover, the less efficient inhibition of KPC-14 by AVI and REL compared to KPC-2 is also reflected in the MIC values we obtained for the combination of ceftriaxone (CRO) with both DBO inhibitors (Table 1), with the KPC-14-producing recombinant clone showing MICs at least two dilutions higher for both CRO-AVI and CRO-REL compared to KPC-2. It is noteworthy to highlight that, until this study, the kinetic behavior of KPC-14 toward relebactam remained unexplored. Regarding avibactam, Oueslati et al. (20) previously compared IC₅₀ for KPC-2 and KPC-14, reporting similar values (230 and 107 nM, respectively). However, in our study, we determined the apparent inhibition constant (*K*_i app_) instead of IC₅₀, providing a more accurate measure of the inhibitor’s affinity for the enzyme. Our results indicate that KPC-14 has a lower affinity for avibactam compared to KPC-2. The discrepancy between our findings and the previously reported IC₅₀ values may be due to the influence of assay’s conditions which might affect IC₅₀ but not *K*_i app_. Additionally, for the IC₅₀ determinations, the authors used ceftazidime as the reporter substrate, which could have affected the results since the concentration used is too close to the corresponding *K*_M_, and therefore steady-state conditions might not be assured.

### Unlike KPC-2, KPC-14 is not able to hydrolyze clavulanic acid

KPC-2 is not effectively inhibited by classical β-lactam inhibitors like clavulanic acid, sulbactam, and tazobactam due to its hydrolytic activity (33, 34). To determine if this behavior is also present in KPC-14, we determined the kinetic inhibition parameters to assess its behavior against clavulanic acid.

We determined the apparent inhibition constant (*K_i_*_app_) of clavulanic acid for both β-lactamases. For KPC-2, the *K_i_*_app_ was expressed as *K*_M_ since its hydrolytic activity for this inhibitor has been previously reported (33, 34). The *K*_M_ obtained for KPC-2 was 80 ± 8 µM, while the *K_i_*_app_ value obtained for KPC-14 was 8.5 ± 1.1 µM. This indicates a 10-fold higher affinity of clavulanic acid for KPC-14 compared to KPC-2. It was not possible to determine the inactivation rate (*k*_inact_) for KPC-14, as the relationship between *k*_obs_ and clavulanic acid concentration was linear rather than hyperbolic.

**Fig 2:**
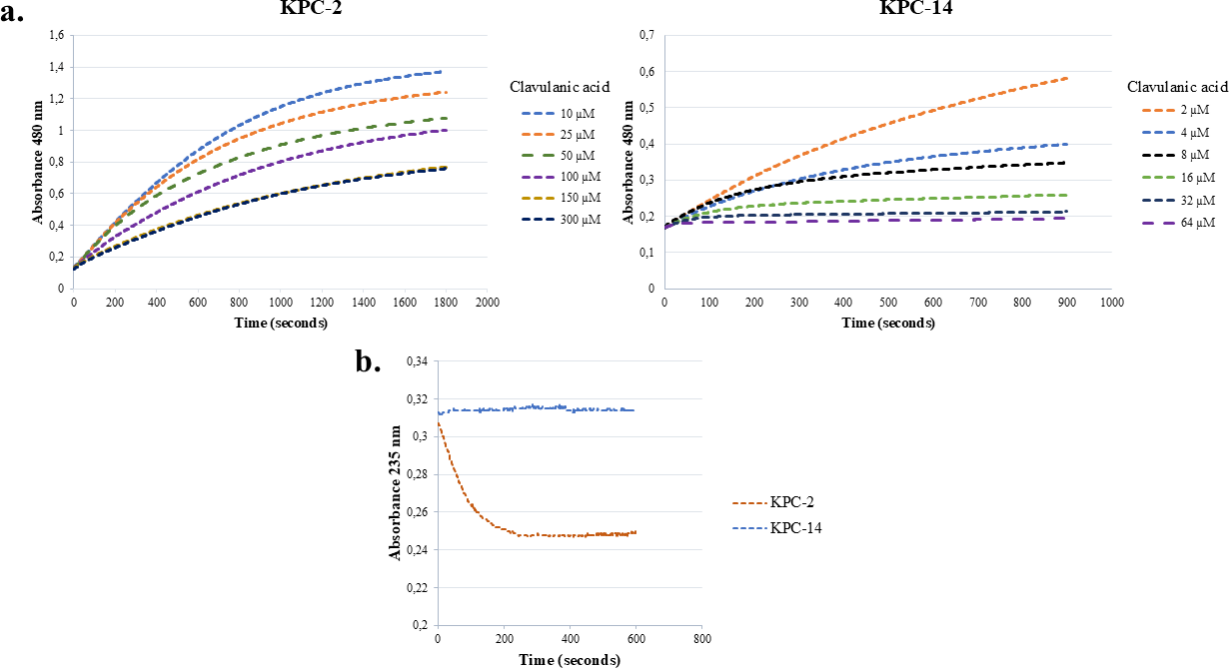
(**a**) Monitoring curves of nitrocefin hydrolysis at 480 nm in the presence of increasing concentrations of clavulanic acid, for KPC-14 and KPC-2. A single representative replicate is shown for each inhibitor concentration tested to simplify the graphic. Compared to KPC-2, the KPC-14 variant is readily inactivated by a concentration of 16 µM of clavulanic acid. (**b**) Monitoring curves of clavulanic acid hydrolysis at 235 nm over time for KPC-14 and KPC-2. A single representative replicate is shown to simplify the graphic.

However, by examining the time courses of nitrocefin hydrolysis in the presence of increasing concentrations of clavulanic acid, it can be observed that a concentration of 16 µM clavulanic acid is sufficient to achieve complete inhibition of KPC-14 activity (Fig. 2). In contrast, higher concentrations of the inhibitor are required to partially inhibit KPC-2, detecting residual hydrolytic activity even in the presence of 300 µM clavulanic acid. The hydrolysis of clavulanic acid was monitored for both β-lactamases at 235 nm, mixing an inhibitor concentration five times above the *K_i_*_app_ (or *K*_M_) and 100 nM enzyme. No hydrolytic activity was detected in KPC-14, while KPC-2 hydrolyzed clavulanic acid with a turnover value (*k*_cat_) of 11.60 ± 0.01 s^−1^.

The analysis of the interaction between clavulanic acid and KPC-14 demonstrated that this variant has a higher affinity for this inhibitor compared to KPC-2 but is also unable to hydrolyze it. These results, in addition to the curves obtained in competitive assays with nitrocefin and increasing concentrations of clavulanic acid, suggest that, unlike KPC-2, KPC-14 is effectively inhibited by clavulanic acid. This kinetic profile, combined with the previously mentioned loss of carbapenemase activity, aligns more closely with an extended-spectrum β-lactamase (ESBL) rather than a serine-carbapenemase like KPC-2. The findings for KPC-14 are consistent with the MIC values for the ceftriaxone-clavulanate combination (Table 1), where the recombinant clone producing KPC-14 showed a fourfold lower MIC compared to KPC-2. They are also in line with observations from other KPC variants, such as KPC-41, -44, -71, and -74 (21, 22, 35, 36), where substitutions in these variants decrease the IC_50_ values for clavulanic acid, indicating increased inhibitory activity of this inhibitor.

### *In silico* modeling of KPC-14 apo enzyme and in complex with ceftazidime and DBO inhibitors

To correlate the observed phenotypic and kinetic differences between KPC-14 and KPC-2, *in silico* modeling of KPC-14 was obtained in its apo enzyme form and in complex with ceftazidime (CAZ), avibactam (AVI), and relebactam (REL).

**Fig 3:**
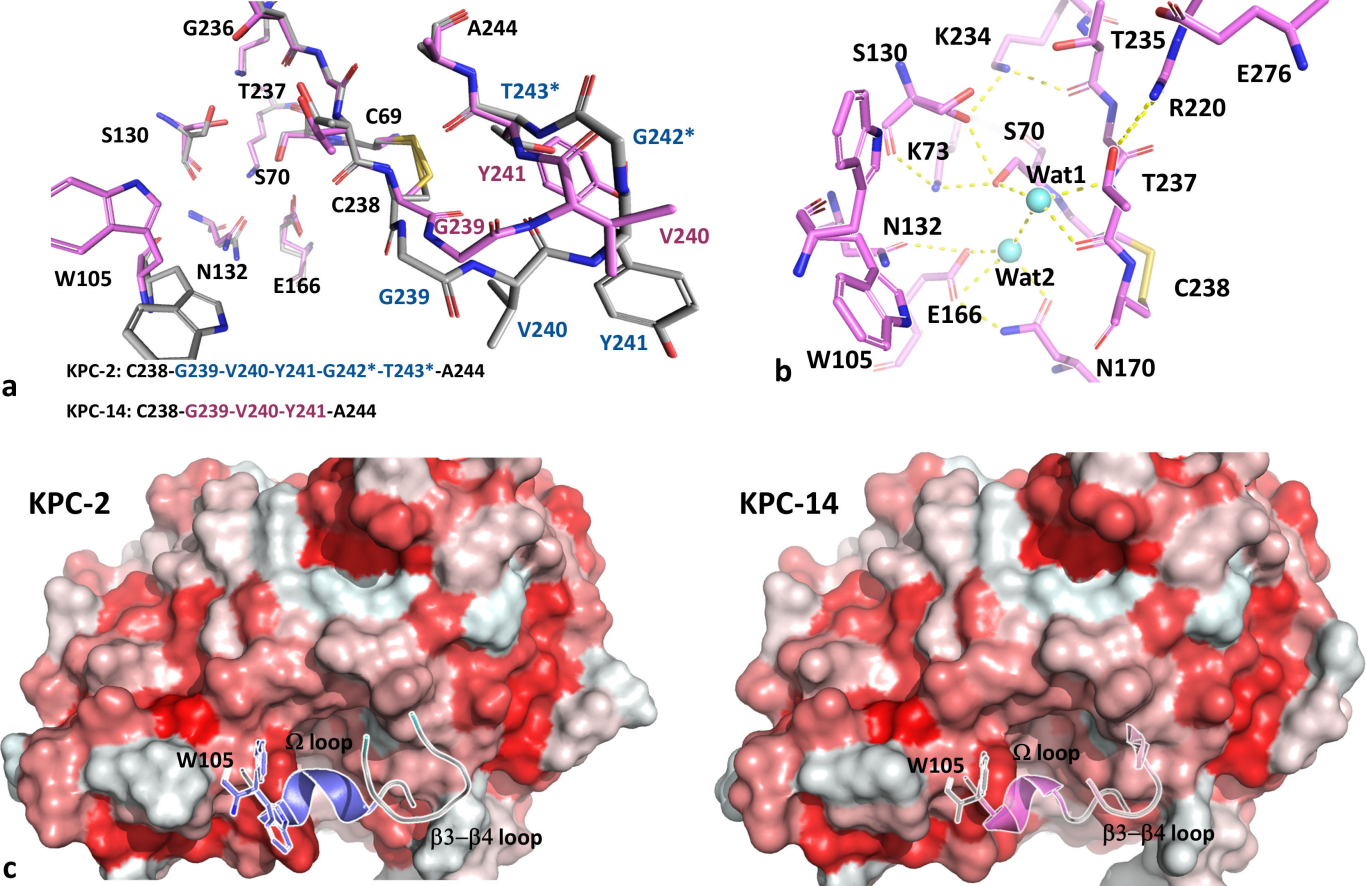
(**a**) Comparative view of the β3-β4 loops of KPC-2 (blue) and KPC-14 (magenta), in which the deletion of G242-T243 (with asterisks) provokes a shortening of the loop, as well as a displacement of residues like V240-Y241 in KPC-14; the β3-β4 sequence is shown in matching colors. (**b**) Details of the active site of KPC-14, showing the main residues involved in the stabilizing hydrogen bonds network, and the probable position of the acylating (Wat1) and deacylating (Wat2) water molecules (light blue spheres). (**c**) Surface view of both KPC-2 and KPC-14: W105, Ω loop, and β3-β4 loop are shown as reference. The surface was colored according to the hydropathy scale, using a gradient from the highest (red) to the lowest (white) hydrophobic content.

The apo KPC-14 model (Fig. 3a) revealed a shortened β3-β4 loop because of the ΔG242-T243 mutation present in this variant. According to this model, the double deletion does not produce significant alterations in the position of relevant residues that coordinate the active site (Fig. 3b), compared to KPC-2 (PDB: 5UL8; RMSD = 0.107). This deletion yields a shortened β3-β4 connecting loop that results in a 5 Å shift of Y241 upward into the protein’s core, probably modifying the hydrophobic content in that interdomain zone (Fig. 3c).

**Fig 4:**
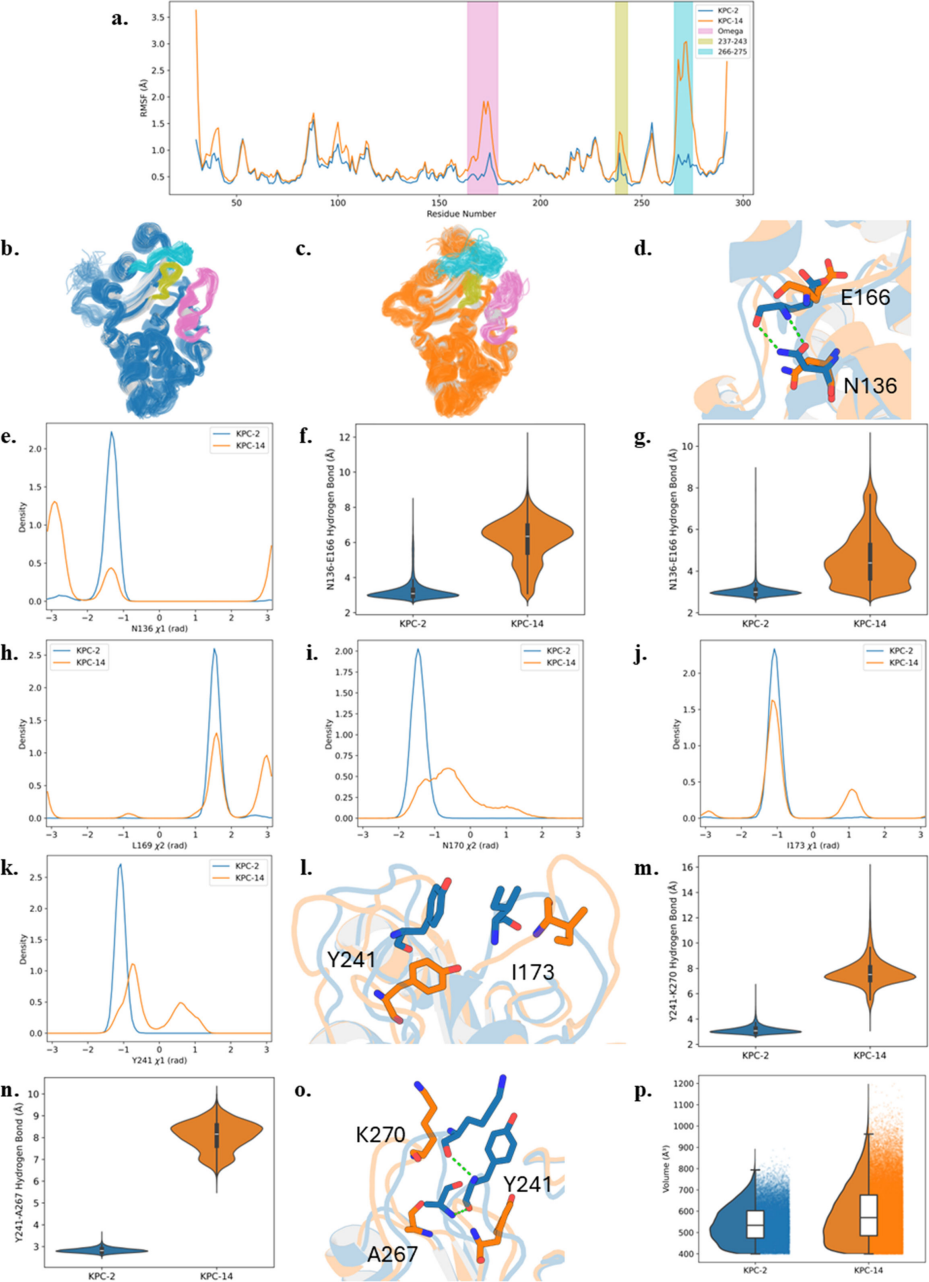
The structures of KPC-2 (blue) and KPC-14 (orange). (**a**) Root mean square fluctuation (RMSF) of KPC-2 and KPC-14. The Ω-loop (pink), the 237–243 loop (olive), and the 266–275 (cyan) are highlighted. (**b**) and (**c**) structural representations of KPC-2 and KPC-14, respectively, illustrating the Ω-loop (pink), the 237–243 (olive), and the 266–275 loop (cyan). (**d**) The hydrogen bond interactions between N136 and E166 in KPC-2. No hydrogen bond is observed between N136 and E166 in KPC-14. (**e**) χ1 dihedral angle density distribution of N136. (**f**) and (**g**) Hydrogen bond distances between N136(ND2)-E166(O) and N136(OD1)-E166(N), respectively. The χ2 dihedral angles density distribution of (**h**) L169 and (**i**) N170. (**j**) Structural conformations of L169 and N170 in KPC-2 and KPC-14. The χ1 dihedral angle density distribution of (**k**) I173 and (**l**) Y241. (**m**) The hydrophobic interactions between I173 and Y241 in KPC-2 and KPC-14. The hydrogen bond distances of (**n**) Y241-K270 and (**o**) Y241-A267. (**p**) Y241 forms hydrogen bonds with K270 and A267 in KPC-2, whereas these interactions are absent in KPC-14. (**q**) Calculated pocket volumes of KPC-2 and KPC-14.

To investigate the regions contributing to the conformational changes, root mean square fluctuation (RMSF) analysis was performed for KPC-2 and KPC-14. The results indicate that the Ω-loop, 237–243 loop, and 266–275 loop exhibit higher flexibility in KPC-14, as evidenced by their elevated RMSF values (Fig. 4a and c). In contrast, these regions in KPC-2 display lower RMSF values, suggesting greater structural stability (Fig. 4b). These observations imply that the double deletion in KPC-14 substantially impacts the stability of these structural regions, particularly the Ω-loop and 266–275 loop. Furthermore, we analyzed the χ1 and χ2 dihedral angles of key residues which are involved in hydrogen bonds or hydrophobic interactions. In KPC-2, N136 preferentially adopts a *gauche(+)* (−60°) conformation (Fig. 4e), enabling the formation of two hydrogen bonds with E166 from the Ω-loop (Fig. 4d, f and g). However, in KPC-14, N136 favors a *trans*(180°) conformation, which disrupts these hydrogen bonds. Additionally, the side chains of L169 and N170 from Ω-loop exhibit greater conformational flexibility in KPC-14 compared to KPC-2. In KPC-2, L169 predominantly adopts a *gauche(*−) (60°) conformation, while N170 favors *gauche(+)* (−60°) conformation (Fig. 4h and i). Conversely, in KPC-14, L169 alternates between two major conformations, *gauche(*−) (60°) and *trans*(180°), whereas N170’s χ2 angle fluctuates between *gauche(+)* (−60°) and *gauche(*−) (60°). This increased flexibility suggests a destabilization of Ω-loop in KPC-14, which could influence its interaction with substrates and inhibitors. Moreover, I173 and Y241 are relatively stable in KPC-2, facilitating a hydrophobic interaction between them (Fig. 4l). In KPC-14, however, this interaction is weakened due to the increased conformation variability of their side chains. Specifically, the χ1 dihedral angle of I173 and Y241 exhibits an additional *gauche(*−) (60°) conformation (Fig. 4j and k), which may reduce the stability of this hydrophobic contact in KPC-14. Overall, these findings indicate that the Ω-loop in KPC-14 is more dynamic than in KPC-2, which is consistent with higher RMSF values observed for this region.

In terms of the stability of 237–243 loop and 266–275 loop, hydrogen bonds play a crucial role (Fig. 4m through o). In KPC-2, Y241 forms hydrogen bonds with K270 and A267, contributing to the structural integrity of these two loops and reducing their flexibility. In contrast, these stabilizing hydrogen bonds are absent in KPC-14, leading to greater mobility and increased structural fluctuations in the 237–243 loop and 266–275 loop. This increased flexibility is reflected in the higher RMSF values observed for these regions in KPC-14, indicating a less stable local structure.

To further assess the impact of these structural differences, the active site volumes of KPC-2 and KPC-14 were measured using MDpocket. The results align with the observed conformational dynamics: the active site of KPC-14 is relatively larger compared to KPC-2 (Fig. 4p), which correlates with the higher flexibility of the Ω-loop, 237–243 loop, and the 266–275 loop in KPC-14. This increased flexibility allows the expansion of active site and potentially enhances its ability to accommodate larger substrates and influence substrate specificity and inhibitor susceptibility.

The crystallographic structure of deacylation-deficient (E166Q) KPC-2 acylated with ceftazidime (PDB: 6Z24) revealed that acylation by CAZ caused significant structural changes involving a disordered Ω-loop (29). The aminothiazole ring of CAZ is located where the N170 residue normally resides in the apo enzyme, explaining why the Ω loop becomes disordered to prevent clashes between the N170 residue and the aminothiazole ring (29). Furthermore, the presence of CAZ in the active site of KPC-2^E166Q^ resulted in the displacement of residues 239 to 243 toward the position of the 266–275 loop (29).

**Fig 5:**
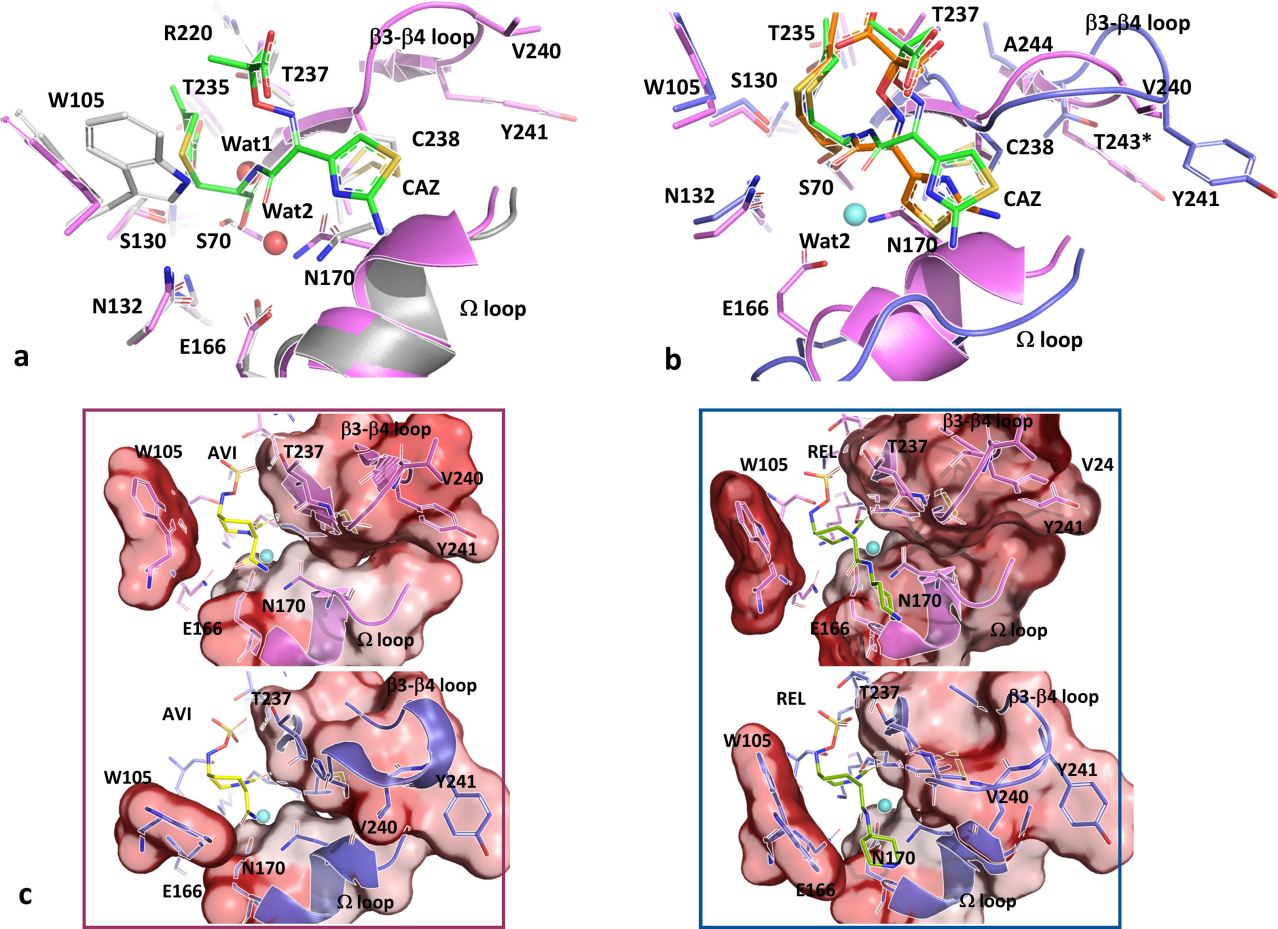
(**a**) Interaction of KPC-14 with CAZ (magenta) compared to the apo KPC-14 (gray). A possible relocation of N170 may be forced by the ceftazidime’s aminothiazole position upon binding of the substrate. (**b**) Compared to KPC-2^E166Q^ (blue ribbon), the aminothiazole ring of CAZ is oriented toward the 237–244 loop compared to KPC-14, avoiding clashes with the Ω-loop and particularly with N170. * Residues are only present in KPC-2. (**c**) Comparative interaction of KPC-2 (blue) and KPC-14 (magenta) with AVI (left panel), and REL (right panel). The hydrophobic content was shown surrounding residues from the Ω loop, β3-β4 loop, and W105, using a gradient from the highest (red) to the lowest (white) hydrophobic content.

According to the *in silico* model of KPC-14 acylated with CAZ, the Ω-loop appears to be ordered, but with a possible relocation of the N170 residue by 1.4 Å forced by the ceftazidime’s aminothiazole position upon binding of the substrate, compared to the KPC-14 apo enzyme model (Fig. 5a). Additionally, in KPC-14 acylated by CAZ, the aminothiazole ring could be oriented further outward from the active site and toward the 237–244 loop compared to KPC-2^E166Q^ acylated by CAZ (Fig. 5b). This would be consistent with the shortened 237–244 loop allowing the aminothiazole ring to be located closer to that loop, avoiding clashes with the Ω-loop and particularly with the N170 residue. This correlates with the ordered Ω-loop and the N170 in its right position toward the active site in the modeling of KPC-14 compared to the displaced orientation of N170 in KPC-2^E166Q^ complex with CAZ (pointing outwards the active site) and a disordered Ω-loop due to the E166Q substitution.

The W105 residue has been implied to play an important role in substrate discrimination in KPC-2 (37). Considering the position of the W105 residue in the model of KPC-14 acylated with CAZ, the presence of this substrate does not alter its position compared to KPC-14 in its apo enzyme form. However, compared to KPC-2 E166Q acylated by CAZ, it adopts an opposite position of almost 180° facing the dihydrothiazine ring of CAZ (not shown), with which it possibly establishes interactions that stabilize this substrate in the active site.

Therefore, *in silico* modeling of KPC-14 acylated with CAZ suggests that the shortened 237–243 loop in this variant allows a less disruptive accommodation of CAZ in its active site compared to KPC-2, which could explain the increased hydrolysis rate of CAZ. A similar observation was obtained in the crystallographic study of the KPC-4 variant, that carries the P104R/V240G mutations, the last substitution located in the same loop as the deletion of KPC-14 (29).

The analysis of KPC-14 modeling in complex with the inhibitors AVI and REL (Fig. 5c and d) did not reveal significant differences in their interaction with relevant active site residues, compared to both the KPC-14 apoenzyme (RMSD value of 0.288 for AVI and 0.318 for REL) and the corresponding crystallographic structures of KPC-2 complexed with AVI or REL (KPC-2/AVI PDB: 4ZBE, RMSD: 0.366; KPC-2/REL PDB: 6QW9, RMSD: 0.338). Future crystallographic studies of KPC-14 acylated with both inhibitors will be necessary to provide accurate structural information that could explain the observed kinetic inhibition behavior for this variant. However, based on the models obtained for this work, we hypothesize that a different hydrophobic content in the active site due to the shortening of the 237–243 loop may interfere with the optimal acylation by AVI and REL in the active site.

### Conclusion

Studying the biochemical characteristics of emerging KPC variants conferring CZA resistance is fundamental to better understanding how their production in clinical isolates can impair the effectiveness of novel antimicrobial treatments.

This investigation provides deeper insights into the biochemical impact of the ΔG242-T243 deletion on the KPC β-lactamase, with a particular focus on its influence on interactions with avibactam and relebactam.

The ΔG242-T243 mutation expands its activity toward efficient hydrolysis of ceftazidime. However, this comes with a trade-off in the activity for penicillins and some cephalosporins while causing a loss of carbapenemase activity. Additionally, the inefficient inhibition by avibactam accompanied by increased hydrolysis of its β-lactam partner ceftazidime explains why the production of this variant compromises the effectiveness of CZA. This correlates with the 16-fold higher MIC for CZA observed in the KPC-14 recombinant clone compared to KPC-2, with the CZA resistance phenotype being further enhanced when KPC-14 production is combined with OmpF deficiency in *E. coli* (data not shown). The decrease in the effectiveness of the CZA combination against KPC-14 can be explained not only by increased hydrolysis of ceftazidime but also by lower affinity and acylation by avibactam, finally resulting in a loss of inhibitory effectiveness by this compound.

The Ω-loop, 237–243 loop, and the 266–275 loop play a crucial role in dynamics. The catalytic mechanism of class A β-lactamases relies on specific conserved residues that facilitate deacylation. The flexibility of the Ω-loop, important for the proper accommodation of substrates, is particularly important in preventing steric clashes between N170 and the aminothiazole ring of ceftazidime (29). The 237–243 loop plays a pivotal role in substrate binding, as its flexibility could determine the extent which the aminothiazole ring of ceftazidime can penetrate the active site. Mutations in this loop, such as T243M or double deletion of G239-V240, enhance the flexibility of the β3 strand, facilitating deeper positioning of the aminothiazole ring within the active site (38). In addition, the structural changes in 266–275 loop are necessary for the repositioning of 237–243 loop and hence preventing the clashes with the aminothiazole ring of ceftazidime (29).

To our knowledge, this is the first study reporting kinetic data on the inhibition of KPC-14 by relebactam, providing novel insights into its potential therapeutic implications. We propose that the lack of efficient hydrolysis of imipenem accompanied by inefficient inhibition by relebactam explains the effective inhibition of the KPC-14-producing clone by the IMR combination. The *in vitro* IMR susceptibility results from our study, along with other reports (39, 40), support that this combination successfully inhibits clones producing different KPC variants. However, further studies on clinical strains are necessary to better determine whether IMR could be considered a promising therapeutic option against microorganisms carrying KPC variants that confer resistance to CZA.

Moreover, it remains unclear whether imipenem or meropenem alone, or in combination with classical β-lactam inhibitors would be effective options for treating infections caused by *K. pneumoniae* harboring KPC variants with ESBL profile. Therapeutic failure with meropenem for treating infections caused by these isolates has been attributed to the presence of mixed subpopulations at the infection site, or the reversion of the mutated *bla*_KPC_ allele back to *bla*_KPC-2_ or *bla*_KPC-3_ (41–44). Most of these studies have focused on KPC variants with substitutions at position D179, which are more likely to revert to the original *bla*_KPC_ gene, as substitutions are generally more likely to undergo reversion compared to deletions or insertions, especially when the latter involve more than one codon. In fact, the reversion of KPC variants with insertion mutations has been associated with a low mutation rate (45).

For this reason, the development of rapid molecular tests capable of detecting the unique phenotypes associated with different types of mutations in the KPC gene would be most welcome in the clinical setting. The ability to distinguish between KPC variants carrying point substitutions versus deletions or insertions is critical for optimizing treatment strategies, particularly those involving novel inhibitor combinations.

Finally, we postulate that deletions in the 237–243 loop could produce CZA resistance through a different mechanism compared to substitutions in the ꭥ-loop.

## MATERIALS AND METHODS

### Bacterial strains and plasmids

The KPC-2 gene was recovered from a clinical *Klebsiella pneumoniae* strain previously characterized in our laboratory (46). *E. coli* TOP10 F´ (Invitrogen, USA) and *E. coli* BL21(DE3) (Novagen, Germany) were used as hosts for transformation experiments, to obtain recombinant clones for antimicrobial susceptibility and overexpression assays, respectively. *Escherichia coli* ATCC 25922 and ATCC 35218 were used as control strains for antimicrobial susceptibility assays.

Plasmid vectors pGEM-T Easy Vector (Promega, USA) and pMBLe (47) were used for general cloning assays and pET24a(+) (Novagen, Germany) for the overexpression of both β-lactamases.

### Chemicals

Nitrocefin was purchased from Cytiva (USA). REL and AVI were provided by R. Bonomo. Imipenem and meropenem were purchased from Merck SA (Argentina). The rest of antibiotics were obtained by donations from the local pharma companies commercializing the drugs.

### Recombinant DNA methodology

The complete *bla*_KPC-2_ gene was amplified from whole DNA from a *Klebsiella pneumoniae* clinical strain by PCR using cloning primers designed to introduce the *NdeI* and *EcoRI* restriction sites: KPC-F-NdeI (5′CATATGTCACTGTATCGCC3′) and KPC-R-EcoRI (5′GAATTCTTACTGCCCGTT3′). The amplified and purified amplicon was cloned into a pGEM-T Easy Vector (Promega, USA), and the resulting construction was transformed into chemically competent *E. coli* TOP10F´ cells. The presence of *bla*_KPC-2_ and restriction sites was verified by DNA sequencing (Macrogen, South Korea).

The resulting pGEM-T/KPC-2 construct was used as a template to obtain KPC-14 by site-directed mutagenesis using the overlap extension method (48). Briefly, combinations of mutagenic and cloning primers were used in PCR reactions to generate two DNA fragments with overlapping ends harboring the mutation. These fragments were subsequently used in a “fusion PCR reaction” to amplify the entire *bla*_KPC-14_ gene with the cloning primers. The mutagenic primers designed were KPC_F1_242–243DEL (5′CCTGCGGAGTGTATGCAAATGACTATGC3′) and KPC_R1_242–243DEL (5′GCATAGTCATTTGCATACACTCCGCAGG3′). The obtained *bla*_KPC-14_ gene was ligated into a pGEM-T Easy Vector (Promega, USA), and the insert was sequenced for verification of the mutagenesis. A proof-reading *Pfu* polymerase (Thermo Scientific, USA) was used in all PCR reactions to avoid errors in the amplifications.

For subsequent cloning, KPC-2 and KPC-14 encoding genes were digested from the corresponding pGEM-T/*bla* construction, and the released fragments were purified and then ligated in the *Nde*I and *Eco*RI sites of pMBLe and pET24a(+) digested vectors. Ligation mixtures were transformed in chemically competent *E. coli* TOP10F´ cells, and recombinant clones were selected in Lysogeny broth (LB) agar supplemented with 20 µg/mL gentamicin or 30 µg/mL kanamycin, depending on whether the constructions were obtained in pMBLe or pET24a(+) vectors, respectively. Recombinant plasmids of the selected clones were extracted and sequenced to verify the identity of *bla* genes and their proper insertion.

### Antimicrobial susceptibility testing

Minimum inhibitory concentrations (MICs) of different β-lactams and combinations with β-lactamase inhibitors were determined by an adaptation of the broth microdilution method by the CLSI (49), as follows. As we used *E. coli* recombinant clones harboring pMBLe/*bla* constructions, where protein production is regulated by isopropyl-β-D-thiogalactoside (IPTG) induction (47), antimicrobial susceptibility assays were performed using Mueller Hinton broth supplemented with 50 µM IPTG. Also, as control strains we included the *E. coli* Top10F’ (used as recipient strain in transformation assays), and the same strain transformed with the empty vector pMBLe, to have a comparison under the same isogenic background.

### Enzyme overexpression and purification

The KPC-2 and KPC-14 purification strategies were designed to purify both β-lactamases in their native state. Recombinant plasmids pET24a(+)/*bla* were transformed into *E. coli* BL21(DE3), and recombinant clones were selected with 30 µg/mL kanamycin. Overnight cultures of recombinant *E. coli* BL21(DE3) producing either KPC-14 or KPC-2 were diluted (1/50) in LB supplemented with 30 µg/mL kanamycin and incubated at 37°C until reaching an optical density (OD) of 0.7–0.8 at 600 nm. The overexpression of β-lactamases was induced with the addition of 0.5 mM IPTG. The induction conditions were optimized for each enzyme: KPC-2 expression was achieved at 37°C for 3 h and KPC-14 induction was carried out at 25°C for 18 h, both with mechanical stirring (180 rpm).

After induction, cultures were harvested by centrifugation (8,000 rpm for 30 min at 4°C), pellets were resuspended with 50 mM sodium phosphate buffer pH 7.0, and cell disruption was achieved by sonication. The obtained crude extracts were centrifuged at 13,000 rpm for 30 min at 4°C, and supernatants were then dialyzed overnight against buffer A (20 mM sodium acetate buffer, pH 5.0) with at least three changes of dialysis buffer. After filtration through 0.45 mm pore-size membranes, clear and equilibrated supernatants were loaded onto a 5 mL HiTrap SP high-performance (HP) column (GE Healthcare Life Sciences, USA) pre-equilibrated with buffer A. Bound proteins were eluted with a continuous gradient (0 to 100%) of buffer B (buffer A + 1M NaCl) and the collected fractions were analyzed in 15% polyacrylamide gels by SDS-PAGE. β-Lactamase activity was tested in all fractions by nitrocefin hydrolysis. Generally, one step of cation exchange chromatography was enough to obtain fractions of purified protein of interest with purity >90%, which was estimated by Coomassie blue staining on 15% polyacrylamide gels. According to the Lambert-Beer law, the protein concentration was determined by UV absorbance at 280 nm. The fractions of the purified enzymes were stored at −80°C for future kinetics assays.

### Kinetics

Steady-state kinetic parameters were determined using a T80 UV/Vis spectrophotometer (PG Instruments Ltd, UK). Each reaction was performed at least in duplicate, in a total volume of 500 µL at room temperature in 50 mM sodium phosphate buffer, pH = 7.0. The steady-state kinetic parameters *K*_M_ and *V*_max_ for different β-lactams were obtained under initial rate as described previously (50), with non-linear least squares fitting of the data (Henri Michaelis-Menten equation) using GraphPad Prism 5.03 for Windows (GraphPad Software, USA) according to equation 1:


(Eq. 1) $$v=(V_{max}\times [S])\;/\;(K_{m}+[S])$$

For low *K*_M_ values, the *k*_cat_ values were derived by the evaluation of the complete hydrolysis time courses as described by De Meester et al. (51). For poor substrates behaving as competitive inhibitors, inhibition constant *K_I_* (as *K_I_*_*obs*_) was determined by monitoring the residual activity of the enzyme in the presence of various concentrations of the antibiotic and nitrocefin as reporter substrate (at a fixed concentration of five times the *K*_M_ for nitrocefin); corrected *K_I_* (considered as apparent *K*_M_) value was finally determined using equation 2:


(Eq. 2) $$K_{I}=K_{I\;obs}\;/\;(1+[NCF]/K_{M(NCF)})$$

Where *K*_M(NCF)_ and [NCF] are the reporter substrate’s *K*_M_ and fixed concentration used, respectively.

For high *K*_M_ values, *V*_max_ could not be reached because initial hydrolysis velocities did not approach enzyme saturation at testable concentrations. In these cases, the slope of the line obtained in initial velocity versus antibiotic concentration plot was considered the second-order rate constant for hydrolysis at steady state (*k*_cat_/*K*_M_), and *K*_M_ values were determined as inhibition constant *K_I_* in competitive assays with nitrocefin as reporter substrate, as above.

The interaction of KPC-2 with avibactam and relebactam was proposed to follow the equation represented in Fig. 1. The formation of the noncovalent complex E:I is represented by *K_I_* (equivalent to *k_-1_/k_1_*). For β-lactamases that acylate very slowly, apparent *K_I_* (*K_I_*_app_) values can approximate the *K_I_* of the inhibitor; otherwise, for β-lactamases with a fast acylation rate, the *K_I_*_app_ approximates the *K_M_* of the enzyme for the inhibitor. The inhibition constants *K_I_*_app_ were determined as reported previously (2, 3), using a direct competition assay under steady-state conditions with nitrocefin as reporter substrate. Initial velocities (*V_0_*) were determined after mixing nitrocefin (at a concentration of five times the *K_M_* for this substrate) with a fixed concentration of enzyme (kept at a nanomolar range) and increasing concentrations of the inhibitor. Inverse initial steady-state velocities (1 */*_V0_) versus inhibitor concentration (I) plot was obtained, and the *K*_I app_ observed was calculated by dividing the value of the y-intercept by the slope of the line. *K*_I app_ values were then corrected by the following equation 3:


(Eq. 3) $$K_{I\;app}(corrected)=K_{I\;app}\;(observed)/(1+([S]/K_{M(NCF)}))$$

For the determination of acylation rate (*k_2_/K*), progress curves were obtained in the same conditions previously mentioned for the *K*_I app_ determination, and then fitted to equation 4 to calculate *k_obs_* values using a nonlinear least-squares with GraphPad Prism 5.03 for Windows (GraphPad Software, USA):


(Eq. 4) $$y=v_{f}\;X\;t+(v_{0}-v_{f})\times (1-e^{-k_{obs}})/k_{obs}+A_{0}$$

For equation 4, *v*_*f*_ is final velocity, *v*_0_ is initial velocity, *t* is time, and *A*_0_ is initial absorbance at *λ* = 482 nm. The data were plotted as *k*_obs_ versus [I], and then *k_2_/K* observed was calculated from the slope of the line according to equation 5, where [I] is the concentration of inhibitor, [S] is the concentration of nitrocefin, and *k_-2_* is the recyclization rate constant:


(Eq. 5) $$k_{obs}=k_{-2}+(k_{2}/K_{obs})\times [I]/(1+([S]/K_{M(NCF)}))$$

Finally, *k_2_/K* value was obtained by correcting the *k_2_/K*_obs_ value considering the concentration and affinity of nitrocefin (equation 6):


(Eq. 6) $$k_{2}/K=k_{2}/K_{obs}\times (1+([S]/K_{M(NCF)}))$$

Previous studies demonstrated that KPC-2 can hydrolyze clavulanic acid (32, 33). To assess if KPC-14 shares the same behavior, *K_I_*_app_ and *k*_inact_ values for clavulanic acid of both enzymes were determined as previously reported (32, 33). The initial velocity (*V*_0_) of clavulanic acid hydrolysis was monitored at 235 nm (32), mixing an inhibitor concentration five times the *K*_M_ determined and 100 nM of enzyme. *V*_0_ obtained under this condition was considered the *V*_max_ and was used to determine the *k*_cat_ for clavulanic acid.

The following extinction coefficients and wavelengths were used: ampicillin (Δ*ε*_235_= –820 M^−1^ cm^−1^), piperacillin (Δ*ε*_235_= –820 M^−1^ cm^−1^), cephalothin (Δ*ε*_273_= –6,300 M^−1^ cm^−1^), ceftriaxone (Δ*ε*_260_= –9,400 M^−1^ cm^−1^), ceftazidime (Δ*ε*_260_= –7,500 M^−1^ cm^−1^), cefepime (Δ*ε*_260_= –10,000 M^−1^ cm^−1^), aztreonam (Δ*ε*_318_= –750 M^−1^ cm^−1^), imipenem (Δ*ε*_300_= –9,000 M^−1^ cm^−1^), meropenem (Δ*ε*_300_= –6,500 M^−1^ cm^−1^), clavulanic acid (Δ*ε*_2350_= –−1,630 M^−1^ cm^−1^), and nitrocefin (Δ*ε*_482_= +15,000 M^−1^ cm^−1^).

### *In silico* modeling of apo and acyl-enzyme complexes

*In silico* modeling of KPC-14 was obtained with Swiss-Model (https://swissmodel.expasy.org/), using the X-ray structure of KPC-2 (PDB 3DW0) as template. Acyl-enzymes of the KPC variants in complex with ceftazidime (CAZ), relebactam (REL) and avibactam (AVI; only for KPC-14) were energy minimized with Yasara (52), using a standard protocol consisting of a steepest descent minimization followed by simulated annealing of the ligand and protein side chains, with the following simulation parameters used: YASARA2 force field, cutoff distance of 6 Å, periodic boundary conditions and water-filled simulation cell. The spatial coordinates of CAZ, REL, and AVI were obtained from the X-ray structures of KPC-2 (E166Q)/CAZ (PDB 6Z24), KPC-2/REL (PDB 6QW9), and KPC-2/AVI (PDB 4ZBE), respectively. All models were visualized with PyMOL 2.4.1 (53).

The trajectories for apo KPC-2 were obtained from the previous study (17). The KPC-14 system was prepared using a protocol identical to KPC-2 as described by Parwana et al*.* (18). Two hundred × 60 ns simulations were run for KPC-14. The trajectories of both KPC-2 and KPC-14 were aligned to their crystal structure conformation via MDAnalysis (54, 55). MDTraj was used to calculate the root mean square fluctuations (RMSF), χ1 and χ2 dihedral angles, hydrogen bonds (56). The hydrogen bond distance was defined as less than 2.5 Å between hydrogen atom and the hydrogen bond acceptor, while the hydrogen bond angle (between donor, hydrogen, and acceptor) was set to be greater than 120° (57). The structures of KPC-2 and KPC-14 were loaded and visualized via PyMol (52). The binding pocket volume of KPC-2 and KPC-14 was measured using MDpocket (58).
